# Modeling Nitrogen Dynamics in a Waste Stabilization Pond System Using Flexible Modeling Environment with MCMC

**DOI:** 10.3390/ijerph14070765

**Published:** 2017-07-12

**Authors:** Hussnain Mukhtar, Yu-Pin Lin, Oleg V. Shipin, Joy R. Petway

**Affiliations:** 1 Department of Bioenvironmental Systems Engineering, National Taiwan University, Taipei 10617, Taiwan; d04622005@ntu.edu.tw (H.M.); d05622007@ntu.edu.tw (J.R.P.); 2 Environmental Engineering and Management Program, School of Environment, Resources and Development, Asian Institute of Technology, Pathum Thani 12120, Thailand; oshipin@ait.asia

**Keywords:** flexible modeling environment, waste stabilization pond, nitrogen dynamic, parameterization, sensitivity, MCMC, GLUE, global uncertainty

## Abstract

This study presents an approach for obtaining realization sets of parameters for nitrogen removal in a pilot-scale waste stabilization pond (WSP) system. The proposed approach was designed for optimal parameterization, local sensitivity analysis, and global uncertainty analysis of a dynamic simulation model for the WSP by using the R software package Flexible Modeling Environment (R-FME) with the Markov chain Monte Carlo (MCMC) method. Additionally, generalized likelihood uncertainty estimation (GLUE) was integrated into the FME to evaluate the major parameters that affect the simulation outputs in the study WSP. Comprehensive modeling analysis was used to simulate and assess nine parameters and concentrations of ON-N, NH_3_-N and NO_3_-N. Results indicate that the integrated FME-GLUE-based model, with good Nash–Sutcliffe coefficients (0.53–0.69) and correlation coefficients (0.76–0.83), successfully simulates the concentrations of ON-N, NH_3_-N and NO_3_-N. Moreover, the Arrhenius constant was the only parameter sensitive to model performances of ON-N and NH_3_-N simulations. However, *Nitrosomonas* growth rate, the denitrification constant, and the maximum growth rate at 20 °C were sensitive to ON-N and NO_3_-N simulation, which was measured using global sensitivity.

## 1. Introduction

A significant number of processes can be used for wastewater treatment prior to its disposal into water bodies [1,2]. A waste stabilization pond (WSP) is an economical, effective and common approach in biological wastewater treatment [3,4,5], especially in tropical climates due to its simple and manageable operation, low capital and operational cost requirements, and favorable climatic factors [6,7,8,9]. WSPs are influenced by stochastically varying environmental factors such as temperature, rainfall, and evaporation [4]. In WSP systems, increased oxygenation attributed to aquatic micro- and macrophytes present during the photosynthesis process has been observed to affect the interfacial oxygen mass transfer across the air–water interface [4]. Numerous studies have indicated that oxygen transfer in WSPs displays high temporal variation, and that mass transfer of oxygen across the air–water interface is a major design parameter [4,10] when modeling WSP systems. Therefore, accurately understanding and modeling the nitrogen dynamics and mechanisms in WSP systems is fundamentally important in assessing performance of, and in designing, WSP systems. WSP systems include complex nitrogen removal mechanisms (i.e., bioassimilation, volatilization, adsorption and chemical precipitation), and long-term adverse effects of nitrogen-rich effluents on water bodies (i.e., eutrophication) [2,11,12,13,14,15,16,17,18].

Many studies have applied mathematical models to simulate nitrogen dynamics and mechanisms in WSP systems [4]. These include simulations of the rate of oxygen absorption incorporating interfacial oxygen by using a lumped parameter model [4], and mass balance equations using system dynamic modeling [11,19]. Recently, various types of models, such as mathematical, deterministic, stochastic, empirical, and dynamic models, have been developed to simulate the process of nitrogen removal and parameter calibration in waste stabilization and high rate algal ponds [13,16,17,20,21,22]. Process-based models have been widely used to examine the dynamics of nitrogen and represent the physical–chemical systems in WSP systems, which usually consist of major parameters that must be calibrated to account for environmental heterogeneity and variations [23,24]. Furthermore, model parameter complexity and the process-based model structure are each sources of uncertainty in model predictions. However, uncertainty estimations can be made when considering how the variability of model parameters affects model simulations. A dynamic model with parameter sensitivity and uncertainty analyses for nitrogen simulation in WSP is a potential model for uncertainty estimations, since the model can deal with dynamic mechanisms of simulated processes both deterministically and empirically [23,24]. The Flexible Modeling Environment developed in R package (R-FME) by Soetaert andPetzoldt [25], provides a flexible and complex modeling environment with modeling algorithms, identifiability analysis [24,26], sensitivity, uncertainty and Monte Carlo analysis [24,26] functionalities. Wu and Liu [27] applied FME with the Soil and Water Assessment Tool (SWAT) model, using an R-SWAT-FME framework to perform parameter identifiability, optimization, sensitivity, and uncertainty analyses. Wu et al. [24] applied FME to simulate the spatial pattern of grain yield production for crops and net primary production (NPP) in their studies. Vigiak et al. [28] used FME in simulating sediment and changes in sediment sources during drought periods. To date, FME-based modeling has not been used for wetland modeling.

In the last decade, a number of sensitivity analysis approaches have been developed and widely applied in hydrological and wetland modeling, such as the Metropolis–Hastings algorithm (MH) [29,30], the Hornberger–Spear–Young (HSY) method of global sensitivity analysis [31,32], the Markov chain Monte Carlo (MCMC) method [17,24,26,33,34], and generalized likelihood uncertainty estimation (GLUE) [24,31,35,36,37]. The above approaches have their advantages, and have been successfully used in parameter sensitivity analysis, optimization, and uncertainty analysis of water quantity and quality models [24,36,38]. GLUE analysis has also been successfully applied in water quantity and quality modeling of wetlands [36,39,40]. Given a specific distribution of inputs, the GLUE analysis procedure estimates the likelihood of all possible outcomes for determinations of behavioral parameter sets of a model [24,36]. The results from any GLUE analysis are an ensemble of behavioral models based on likelihood values [31]. Integrating GLUE analysis into R-FME can therefore provide additional uncertainty analysis for modeling uncertainty in FME [25,41].

The objective of this study is to develop a dynamic simulation model for WSP by using R-FME with MCMC in order to evaluate the nitrogen dynamics and perform a comprehensive modeling analysis, with a focus on parameter optimization, local sensitivity, parameter global sensitivity, and uncertainty analysis. GLUE analysis was integrated into the R-FME model to optimize model parameters and simulate the concentrations of ammonia nitrogen (NH_3_-N), organic nitrogen (ON-N), and Nitrate (NO_3_-N) in the study WSP. Global sensitivity and parameter sensitivity analyses for the simulations were performed using MCMC and GLUE.

## 2. Materials and Methods

**Figure 1:**
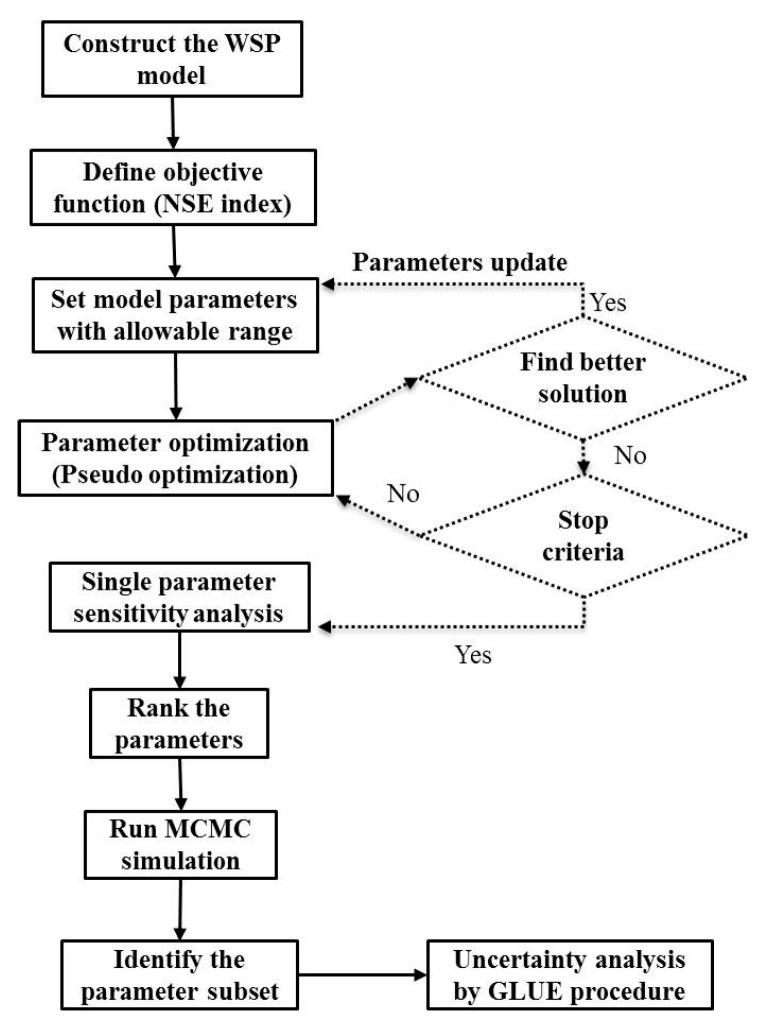
Flowchart of the model development process.

Figure 1 illustrates the WSP model development process including calibration, validation, local and global sensitivity analysis, and GLUE analysis using R-FME.

### 2.1. Site and Experiment Design

**Figure 2:**
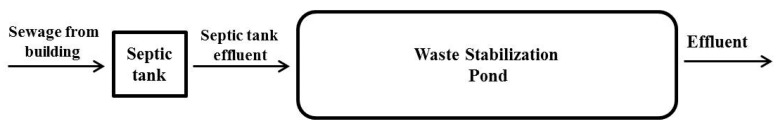
Layout of septic tank–waste stabilization pond system for the treatment of wastewater from a residential building.

Observed data from a pilot-scale WSP system for treating septic tank effluent was used for model calibration and validation. The experiment measured the nitrogen removal capacity of ponds designed for flood control on a university campus in Thailand (at coordinates: 14°04′46.00″ N, 100°36′50.51″ E). A hydraulic retention time of 10 days was maintained in the pond, and the average influent nitrogen concentration was 10.24 mg/L, 16.8 mg/L, and 1.03 mg/L for ON-N, NH_3_-N, NO_3_-N, respectively. The pond length, width, and depth measured 20.5 m, 1.2 m, and 0.80 m, respectively (Figure 2).

Water samples were collected and monitored weekly for one year during 10:00–11:00 am of the sampling day from June 2014 to July 2015. Influent and effluent water samples from each of the systems (400 mL each) were analyzed for total Kjeldahl nitrogen (TKN), ammonia nitrogen (NH_3_-N and NH_4_-N), nitrite, and nitrate nitrogen (NO_x_-N). Temperature, dissolved oxygen, and pH were measured at each sampling location. All measurements were conducted according to methodology endorsed by the Standard Methods for the Examination of Water and Wastewater [42]. Statistical procedures and one-way analysis of variance (ANOVA) to evaluate the significance of the differences in influent and effluent were carried out using Statistical Packages for Social Scientists (SPSS version 12.0).

### 2.2. R-FME Functions Adopted in This Study

R package FME [25,41] was developed to perform comprehensive analysis for inverse modeling with optimization techniques (“Nelder-Mead”, “BFGS”, “CG”, “LBFGS-B”, “SANN”, “Newton”, “Pseudo”), parameter identification, sensitivity analyses, and Monte Carlo analyses. Detailed descriptions of FME functions were introduced by Soetaert and Petzoldt (2010) [25] and are available on the R Archive Network at http://CRAN.R-project.org/package5FME. This study developed WSP models with R-FME, as described in Section 2.4. We considered three target variables, including NH_3_-N, ON-N, and NO_3_-N, and multiple parameters with allowable ranges for simulating nitrogen dynamics in a WSP. Two functions within R-FME, “sensFun” and “Collin”, were used to estimate local sensitivities and parameter identifiability of nine parameters. The sensFun function used initial parameter values and an allowable parameter range based on the sum of squared residuals and standard deviation of the observed data in order to calculate the local sensitivity of parameters for model output. The Collin function provided collinearity index values to identify the corresponding parameter subsets. Table S1 lists the descriptions of the R-FME functions used in this study.

### 2.3. GLUE Analysis

Developed by Beven and Binley [35], GLUE analysis executes numerous model runs with generated random parameter values from a priori probability distributions [35,37,43]. Each run is defined as “non-behavioral” when its likelihood value is less than a defined threshold. The GLUE analysis samples from an assumed probability model of uncertain parameters, and estimates the uncertainty in model outputs as a function of likelihood [44]. Detailed discussion of the GLUE method can be found in Beven and Binley (1992, 2014) [31,35]. In this study, several steps of the general GLUE method were adapted for our investigation. The simulated variables of the nitrogen removal model (ON-N, NH_3_-N, and NO_3_-N) were compared with observed data using the likelihood functions of the Nash–Sutcliffe coefficient of efficiency (NSE) [38,45]. A likelihood value threshold was used to evaluate non-behavioral and discarded parameters sets. The NSE is used as a goodness-of-fit criterion and as the likelihood measure, defined as follows:

(1) $${L\;(\theta }_{i}|Y)=(1-\sigma_{i}{}^{2}/\sigma_{\mathrm{obs}}{}^{2}),$$

where ${L\;(\theta }_{i}|Y)$ denotes model simulation likelihood measures for a set of observations, $\sigma_{i}{}^{2}$ denotes the associated error variance, and $\sigma_{\mathrm{obs}}{}^{2}$ denotes the observed variance. In this study, MCMC was used to generate 5000 parameter sets (within the range of −10% to +10%), whereby each parameter set consisted of nine parameters (Table 1 and Table 2). Uniform distribution was assumed, since an unknown a priori distribution is also assumed. Parameter ranges are based on published values from established studies (Table 2).

### 2.4. Model Construction

A WSP system dynamic model was developed using the R-FME package. R-FME functions were used to write the set of differential equations, and were solved numerically by using the “deSolve” package. It was assumed that the study WSP is completely mixed and acts as a single continuous stirred tank reactor [46]. Mass balanced equations for organic nitrogen (ON-N), ammonia nitrogen (NH_3_-N), and nitrate nitrogen (NO_3_-N) concentrations are as follows [21,46]:

(2) $$\frac{d\;\left(\mathrm{Org}-N\right)}{dt}=\;\frac{Q_{i}}{V}{\left(\mathrm{Org}-N\right)}_{i}-\;\frac{Q_{e}}{V}\;\left(\mathrm{Org}-N\right)-r_{m}-r_{s}+r_{1}+\;r_{2},$$

(3) $$\frac{d\;\left({\mathrm{NH}}_{3}-N\right)}{dt}=\;\frac{Q_{i}}{V}{\left({\mathrm{NH}}_{3}-N\right)}_{i}-\;\frac{Q_{e}}{V}\;\left({\mathrm{NH}}_{3}-N\right)-r_{1}+r_{m}-r_{v}-\;r_{n},$$

(4) $$\frac{d\;\left({\mathrm{NO}}_{3}-N\right)}{dt}=\;\frac{Q_{i}}{V}{\left({\mathrm{NO}}_{3}-N\right)}_{i}-\;\frac{Q_{e}}{V}\;\left({\mathrm{NO}}_{3}-N\right)+r_{n}-r_{2}+r_{d},$$

where *Q_i_* and *Q_e_* are influent and effluent flow rate (m^3^/d), respectively; *V* is volume of the pond (m^3^); *r_n_* is nitrification rate (mg/L.d); *r_d_* is denitrification rate (mg/L.d); *r_m_* is mineralization rate (mg/L.d), *r_s_* is net loss of Org-N (mg/L.d); *r_v_* is volatilization rate (mg/L.d); *r*_1_ is uptake rate of NH_3_-N by microorganisms (mg/L.d); and *r*_2_ is uptake rate of NO_3_-N by microorganisms (mg/L.d).

Table 1 lists mass balance reaction rate models used in this study and each model’s process descriptions. This study considers biomass as proportional to organic nitrogen, represented as Org-N [11,13]. Organic nitrogen converts to ammonia through a mineralization process (*r_m_*) that can be simulated by first-order kinetics as a function of ON-N concentration [47]. Nitrification (*r_n_*) of NH_3_-N, where ammonia nitrogen is converted to nitrate through biological oxidation, is modeled as a single step process that is a function of temperature, dissolved oxygen, and pH [13]. The half saturation constant for Nitrosomonas $\left(K_{n}\right)$, the temperature dependence factor $\left(C_{T}\right)$, and Nitrosomonas growth-inhibiting factor for pH $\left(C_{pH}\right)$ are computed from Downing’s model (Table 1) [13]. In the nitrification rate function, Y_n_ represents the mass of *Nitrosomonas* as per unit mass of substrate (mg/mg) and µ_n_ represents the *Nitrosomonas* growth rate. A rate-limiting factor for *Nitrosomonas* growth is pH, which is corrected by *C_pH_*. When pH is below 7.2, the presence of free ammonia reduces the growth of nitrifying bacteria that react, according to equations shown in Table 1. However, when pH is greater than 7.2, no significant inhibition occurs for nitrifying bacteria and *C_pH_* = 1 [13]. Nitrification is also influenced by temperature. An exponential function is used to describe the temperature correction factor (*C_T_*) when *T*_o_ equals the reference temperature (15 °C).

Volatilization of ammonia through ammonia stripping is dependent on pH, pond depth (d), temperature (*T*), and ammonia concentration in the WSP, and is modeled using equations proposed in recent studies [47,48]. Ammonia and nitrate convert into organic compound mostly by autotrophs [11,49]. Photosynthesis is the main means by which autotrophs produce organic compounds and oxygen, which is done by consuming carob dioxide, water, and nutrients from their surroundings. Regarding microorganism growth (*r*_1_, *r*_2_), microorganisms consume ammonia nitrogen as well as nitrate nitrogen, but prefer ammonia to nitrate for cell synthesis [11]. Preference factors *p*_1_ and *p*_2_ are therefore introduced to represent the ammonia and nitrate uptake by microorganisms, respectively.

In the WSP system, non-biodegradable organic nitrogen settles into sediments and is considered to be the permanent removal of organic nitrogen from the influent. Previous studies showed that 10 days of hydraulic retention time was sufficient for proper sedimentation in ponds and wetlands [50,51] where the sedimentation coefficient value (Rs) varies between 0.001 to 0.1 d^−1^ [46,47]. Net loss of nitrogen to sediment (*r_s_*) in WSP is calculated as a function of ON-N concentration where the sedimentation coefficient (Rs) was obtained from model calibration. Denitrification is performed primarily by heterotrophic bacteria. The rate-limiting factors for denitrification are temperature, nitrite, and nitrate concentration [11]. However, nitrite is not modeled in this study due to very low concentrations in the influent (less than 0.1 mg/L). Nitrification and denitrification within aquatic systems can occur in sediments, and have been studied extensively [52,53]. In treatment ponds and wetlands, however, sediments play a negligible role in the system’s nitrification and denitrification processes [11]. Moreover, if the WSP is working properly, denitrification (*r_d_*) may occur near the bottom layer of the system and is modeled using first-order Arrhenius kinetics [13].

The performance of the proposed model was evaluated by comparing its simulated output with measured outflow concentrations of NH_3_-N, ON-N, and NO_3_-N using NSE values. The “Pseudo” optimization technique with 500 iterations was used to obtain the optimal parameter values, and was based on the first six months of weekly observations of the three target variables between June 2014 and December 2014. Pseudo optimization was employed to bring the algorithm near vicinity, after which the MCMC algorithm was used to maximize the NSE value. Iterations were stopped upon criteria satisfaction (NSE > 0.5). The model was then validated using the observed data from the subsequent six months, January–July 2015.

## 3. Results

### 3.1. Local Sensitivity and Parameter Analyses

Table 2 summarizes the parameter ranking results of the dimensionless parameter sensitivity values. A value with high absolute sensitivity index (L1) represents high importance and ranking. Compared to other narrow range parameters, the L1 results show that θ, K_1_, and K_2_ are ranked 1st, 2nd, and 3rd, respectively, followed by Rs, µ_n_, and µ_max_. Parameter identifiability is important for parameter estimation to examine the degree of near-linear dependence of the sensitivity function of all possible parameter subsets. Sensitivity measurements estimate the identifiability of a parameter on an individual basis, while collinearity estimates a parameter subset’s compensation in joint estimation. A collinearity index value below 15 indicates that the corresponding parameter subsets are highly identifiable. In this study, the selected parameters were highly identifiable as the collinearity index (Figure S1) for various parameters was always less than four. The collinearity index for the selected nine parameters are shown in Figure S1.

### 3.2. Parameter Optimization

**Figure 3:**
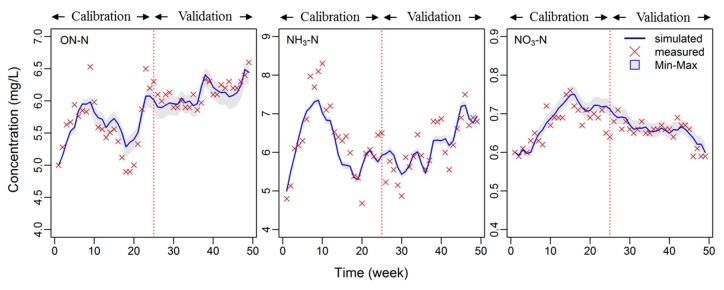
Weekly time series of observed and simulated ammonia nitrogen (NH_3_-N), organic nitrogen (ON-N), and Nitrate (NO_3_-N) during a one-year calibration and validation period from 2014–2015. Sensitivity ranges of weekly ON, NH_3_, and NO_3_ were generated with the Markov chain Monte Carlo (MCMC) application. Gray shading indicates minimum and maximum model responses at each time step.

Figure 3 shows the weekly time series of three observed and simulated variables (i.e., ON-N, NH_3_-N, and NO_3_-N) during both the calibration and validation periods. The calibrated parameter values are listed in Table 2. It was observed that many of the parameters were either identical to or within the range of those values obtained by other researchers. Results show that the optimized θ (Arrhenius constant) and K_2_ (“Ammonium. half sat. const.”) are very close to their lower bound (1.01). This value is in line with most other applications. Some literature reported a θ value of 1.02 for waste stabilization ponds [14,47] while Wang et al. [43] reported a higher θ value (1.04) when simulating pollution removal in a constructed wetland, than those found in the current study (1.01).

### 3.3. Model Performance Evaluation

A significant difference in the nitrogen concentration of influent and effluent (*p* < 0.001) was observed. Table S2 shows ANOVA test results for ON-N concentrations in influent and effluent. Similar results for NH_3_-N and NO_3_-N are not shown. Mean removal efficiency for ON-N, NH_3_-N, and NO_3_-N was 46.3%, 62.8%, and 61.4%, respectively. However, there was no significant difference (*p* > 0.1) in removal efficiency during calibration and validation periods, since there is minimal seasonal variation in tropical climates [2,9].

Table 3 summarizes the statistical analysis for the three variables used in this study for both calibration and validation. The results show that the simulations of the three targeted variables (ON-N, NH_3_-N, and NO_3_-N) were quite close and fit well with their corresponding observations for both calibration and validation periods. The NSE for NH_3_-N and ON-N were 0.69–0.70 and 0.53–0.66, respectively, while the corresponding correlation constant (*r*) values for both were 0.83–0.87 and 0.76–0.84, respectively. The NSE value for NO_3_-N, however, was lower when compared to NH_3_-N and ON-N.

### 3.4. Uncertainty and Global Sensitivity Analyses

Model uncertainty for the WSP model, with an allowable parameter range, was estimated with R-FME-based uncertainty analysis and GLUE analysis. The Bayesian–MCMC method has been widely used in uncertainty analysis [59]. Parameter uncertainty, with an allowable parameter range, was quantified with the R-FME “modMCMC” function.

**Figure 4:**
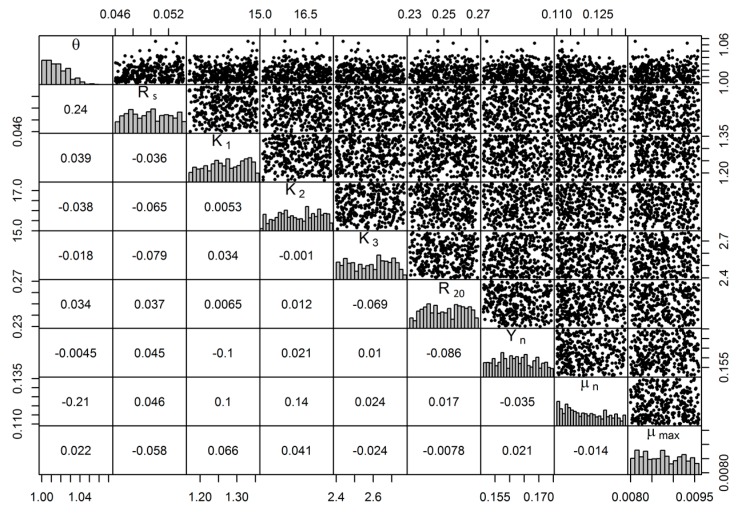
Pairs plot of the Markov chain Monte Carlo (MCMC) samples for the nine parameters. Note: Top is the pairwise relationship; bottom is the correlation coefficient; diagonal is marginal distribution.

Figure 4 shows the MCMC sample pair plot based on 5000 runs, with a scatterplot matrix of coefficients and marginal frequency distributions for each parameter in top, bottom, and diagonal plots [23]. The results show that the parameters (Arrhenius constant (θ) and sedimentation constant (R_s_)) are related, because both are closely linked to nitrogen simulation in ponds [43]. However, there is no positive correlation between other parameters. Regarding parameter identifiability, the correlation coefficient values among parameters are small (<0.25), so the parameter is identifiable [33].

**Figure 5:**
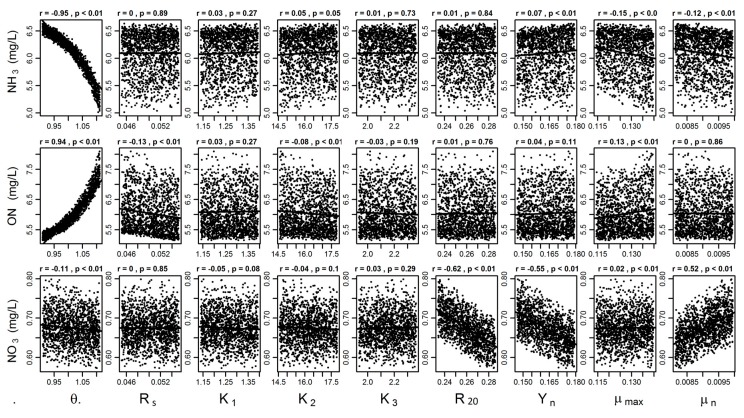
Global sensitivity analysis results of ammonia nitrogen (NH3-N), organic nitrogen (ON-N), and nitrate (NO_3_-N) for the one-year calibration and validation period (2014–2015), as a function of uniform distribution of the parameter values.

The “modCRL” (global sensitivity analysis) function was used to estimate the effect of a uniform parameter distribution on the mean model output in their allowable parameter range. Figure 5 shows the results of the modMCMC global sensitivity analysis of ammonia nitrogen (NH_3_-N), organic nitrogen (ON-N), and nitrate (NO_3_-N) for the one-year calibration period (2014–2015), as a function of uniform distribution of the parameter values. The results indicate that the MCMC parameter values for each iteration are well mixed within upper and lower ranges of allowable parameter values. There is a clear linear and negative relationship between NH_3_-N and θ (Arrhenius constant) (*r* = −0.95, *p* < 0.01), but a positive relationship between ON-N and θ. However, there is a near-linear and negative relationship between output variable NO_3_-N and the parameters Y_n_ (yield coeff. *Nitrosomonas*) and R (denitrification constant).

**Figure 6:**
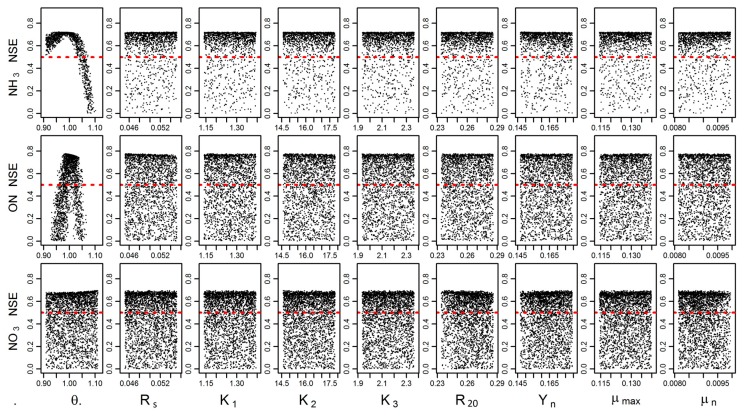
Scatter plot of Nash–Sutcliffe coefficient of efficiency (NSE)-sensitive parameters based on generalized likelihood uncertainty estimation (GLUE) method for ammonia nitrogen (NH3-N), organic nitrogen (ON-N), and Nitrate (NO_3_-N). Note: dots represent the Nash–Sutcliffe coefficient value for each iteration; red horizontal line indicates the NSE minimum criteria.

**Figure 7:**
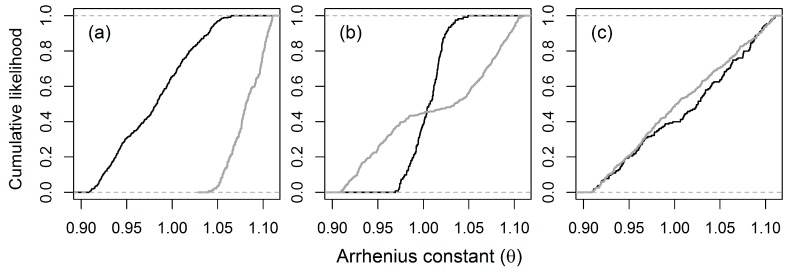
Cumulative density functions of behavior and non-behavior solutions based on the NSE likelihood functions for (**a**) ammonia (NH_3_-N), (**b**) organic nitrogen (ON), and (**c**) nitrate (NO_3_-N). Black indicates behavioral likelihood (NSE ≥0); gray indicates non-behavioral likelihood (NSE <0).

In GLUE analysis, a range of model responses was estimated to simulate the proposed model output using all behavioral parameter sets, which are either accepted as behavioral (likelihood value ≥0) or rejected as non-behavioral (Likelihood value <0), and are a function of its goodness of fit. Figure 6 shows the results of the GLUE analysis, where dots represent the Nash–Sutcliffe coefficient value for each iteration of MCMC within the parameter range, and the red horizontal line indicates the NSE minimum criteria. The scatter plots also revealed the feasible values of all parameter ranges within the calibration values [31]. The parameter range and sensitivity of nine parameters individually sensitive to ammonia nitrogen (NH_3_-N), organic nitrogen (ON-N), and Nitrate (NO_3_-N), are associated with likelihood values. GLUE sensitivity analysis results indicate that sensitivity parameters included θ (Arrhenius constant) in simulating NH_3_-N and ON-N, and that slight or no sensitivity is observed for the remaining parameters set [17]. Figure 7 shows cumulative density functions of behavior and non-behavior solutions based on the NSE likelihood functions for the sensitive parameter (Arrhenius constant). The cumulative distributions represent the behavioral and non-behavioral distributions, which show the NSE ≥0 and NSE <0, respectively. The results show that the behavioral and non-behavioral distributions for NO_3_-N are close to each other when compared to ON-N and NH_3_-N.

## 4. Discussion

### 4.1. Parameter Optimization and Model Performance

The accuracy of predicted organic nitrogen concentration in effluent was improved by increasing the calibrated value of the sedimentation constant (Rs) to 0.050, instead of using the value reported in literature (0.015) [47]. K_1_, K_2_, and K_3_ represent the Nitrosomonas half-saturation constant, ammonium half-saturation constant, and nitrate half-saturation constant, respectively [21]. K1 was increased from 0.15 to 1.26 to improve the simulation of ammonia and nitrate. The calibrated value for K3 was the same as the initial value (2.0), and likely caused by high proportions of NH_3_-N in influent, since microorganisms tend to prefer ammonia over nitrate, and nitrate consumption does not occur until the ammonia is almost completely consumed [60]. In this study, the calibrated R_20_ value was within the “literature range” obtained by other researchers (0.0–1.0) [47,56]. Yet, the simulation of pollution removal in a constructed wetland by Wang et al. [43] reported an even higher value (3.74) than those found in the current study (0.26). Model performance can be improved by further reducing the θ value (Arrhenius constant); however, in this study, real-world application was prioritized over obtaining a better parameter value.

Similar to results from reviewed literature, this study observed that the major pathway of nitrogen removal in WSP could be ammonia volatilization [61,62] influenced by environmental factors, macro-microphyte abundance, and influent NH_3_-N concentrations. In the current study, volatilization played only a negligible role in nitrogen removal. Similar to results reported by Valero and Mara [63], the ammonia volatilization process accounted for only 3% of the total nitrogen removal in the WSP system.

Some studies showed that nitrification-denitrification is not likely to be a major pathway for nitrogen removal in pond systems. However, mineralization, nitrogen uptake by microorganism growth, and ammonia stripping are the most important mechanisms in WSP systems operating at various hydraulic retention times [64]. Moreover, microorganisms are assumed to consume NO_3_-N only when NH_3_-N is not available. This may explain the low NSE value for NO_3_-N. Nutrient removal in algal-based wastewater treatment systems could be affected by the interaction of algae with other microorganisms, such as bacteria [17,65,66]. Increased pH and inhibitory metabolites attributed to algae presence has been observed to affect bacterial activity [66]. On the other hand, algae can be damaged by the soluble cellulose enzymes released by some bacterial species. The interactions between algae and bacteria are complex and will be influenced by environmental conditions [67]. Further studies are therefore needed to understand the interactions between algae and bacteria to improve our understanding about nitrogen removal dynamics in WSP systems.

### 4.2. Uncertainty and Global Sensitivity Analyses

The parameters with allowable ranges were analyzed with MCMC and GLUE procedures to estimate uncertainty in nitrogen simulations. The results indicate that both methods perform similarly in terms of determining best parameter values. The NSE index scatter plots provide feasible values within the parameter range over the most calibration values. However, the parameter sensitivity results estimated by MCMC and GLUE were significantly different. MCMC sensitivity analysis results indicated that sensitivity parameters included θ, R_20_, Y_n_ and µ_n_. However, GLUE sensitivity analysis indicated that θ was the only sensitive parameter in model simulations, while no sensitivity was observed for the remaining parameters set. In GLUE uncertainty analyses, parameter sensitivity is subject to certain threshold values that separate the total parameter set into behavioral and non-behavioral parameters as a function of goodness of fit and measured by comparing the model simulation results and observations [39]. In this study, all parameter sets that resulted in NSE <0 were discarded. Due to this, MCMC sensitive parameters (R_20_, Y_n_ and µ_n_) were not variants of GLUE behavioral parameters (NSE >0), and cumulative distribution for behavioral solutions always equaled one for a given parameter range. Likelihood functions with threshold values have a significant effect on the parameter uncertainty estimation. It is recommended that modelers choose the best option based on the factors involved in their decision-making approach. The *Nitrosomonas* species accomplishes an important function of aerobic ammonia oxidation [17,18]. Ochoa et al. [17] indicated that the yield factor for heterotrophic bacteria contributed to the simulation of NO_3_. In this study, R_20_, Y_n_, and µ_n_ are sensitive in MCMC analysis of NO_3_ concentration simulations. Though sedimentation of organic nitrogen is a major nitrogen removal mechanism reported by Møller et al. [16], in this study, Rs (sedimentation constant) was only slightly sensitive in the MCMC analysis of ON concentration simulations.

Nash and Stucliffe’s efficiency measure with a threshold value has been widely used to identify a set of behavioral models [31]. Parameter sensitivity can also be described by orientation to and gradation of the likelihood distribution of functions. A parameter will be highly sensitive to a likelihood function if cumulative distributions are far apart from each other. On the other hand, a parameter will be less, or not sensitive, to a given set of likelihood functions if cumulative distributions of likelihood functions are close to each other [39]. Differing from the results of Møller et al. [16] but similar to that of Ochoa et al. [17], this study’s sensitivity analysis results show that the Arrhenius constant is highly sensitive to ammonia (NH_3_-N) and organic nitrogen (ON) simulation, as behavioral and non-behavioral distribution tends to be far apart. Alternatively, the closely distributed behavioral and non-behavioral solutions show that the Arrhenius constant is not sensitive to nitrate (NO_3_-N) simulation. These results reiterate the need for the careful selection of likelihood functions, due to their strong influence on parameter sensitivity [31,68].

## 5. Conclusions

This study presented an approach that integrates R-FME with MCMC and GLUE in simulations of concentrations of ON-N, NH_3_-N, and NO_3_-N at a pilot WSP system in Thailand. Using the proposed approach, WSP models were developed in R-FME to successfully derive: an optimal parameter set; global sensitive parameters for ON-N, NH_3_-N, and NO_3_-N concentrations; and delineated sensitive sets of parameters from GLUE behavior sets based on predefined thresholds of model performance. The major advantages of using an FME-GLUE approach include its modeling comprehensiveness, which incorporates parameter sensitivity analysis and global sensitivity analysis for application to other physical-chemical processes. The results indicate that the Arrhenius constant is the parameter sensitive to simulated ON-N concentrations, the denitrification constant, and the yield coefficient. Further, the Arrhenius constant was the only parameter sensitive to model performances of ON-N and NH_3_-N simulations. The *Nitrosomonas* growth rate, denitrification constant, and the maximum growth rate at 20 °C were sensitive to simulated concentrations of ON-N and NO_3_-N, which was measured using global sensitivity. The proposed approach is a potential tool to indicate and quantify the model parameter uncertainty of various target variables for WSP systems. Additionally, our proposed approach, which uses R functions, is adaptable for users to develop their own models or additional functions based on model structures.

## Figures and Tables

**Table 1:** Mass balance reaction rate models.

Process	Model	Source Literature
Mineralization	$r_{m}=0.002T\;\times \left(\mathrm{Org}-N\right)$	[47,54]
Nitrification	$r_{n}=\;\frac{{µ}_{n}}{Y_{n}}\;\left(\frac{NH_{4}-N}{K_{n}+\;NH_{4}-N}\right)\;\left(\frac{\mathrm{DO}}{K_{1}+\mathrm{DO}}\right)\;C_{T}\;\times \;C_{pH}$ $K_{n}=\;10^{\left(0.051(T-1.58\right)},C_{T}=\mathrm{Exp}\;(0.098\;\times \left(T-15\right)$ $\begin{array}{cc} \mathrm{IF}\;\mathrm{pH}<7.2\;THEN & C_{\mathrm{pH}}\\ & =1-0.833\left(7.2-\mathrm{pH}\right){\;\mathrm{ELSE}\;C}_{\mathrm{pH}}\\ & =1 \end{array}$	[54]
Sedimentation	$r_{s}=\;R_{s}\;\times \;\left(\mathrm{Org}-N\right)$	[46,47]
Denitrification	$r_{d}=$ R_20_ $\theta^{\left(T-20\right)}$ NO_3_-N	[46]
Volatilization	$r_{v}=\;\frac{NH_{3}-N\;\times 0.0566\;\times Exp\left(0.13\left(T-20\right)\right)}{d\;\times \;\left(1+10^{\left(10.5-0.03T-pH\right)}\right)}$	[46]
Microorganisms growth 1	$r_{1}\;={\;µ}_{\max\;20{\;\theta }^{\left(T-20\right)\;}}\left[\frac{{\mathrm{NH}}_{3}-N}{K_{2}+{\;\mathrm{NH}}_{3}-N}\right]\;\left(\mathrm{Org}-N\right)\times P1$	[13,55]
Microorganisms growth 2	$r_{2}\;={\;µ}_{\max\;20{\;\theta }^{\left(T-20\right)\;}}\left[\frac{{\mathrm{NO}}_{3}-N}{K_{3}+{\;\mathrm{NO}}_{3}-N}\right]\;\left(\mathrm{Org}-N\right)\times P2$	[55]

Note: *r_m_* = mineralization rate (mg/L.d), *r_d_* = denitrification rate (mg/L.d), *r_n_* = nitrification rate (mg/L.d), *r_s_* = net loss of Org-N (mg/L.d), *r_v_* = volatilization rate (mg/.·d), *r*_1_ = uptake rate of NH_3_-N by microorganisms (mg/L.d), *r*_2_ = uptake rate of NO_3_-N by microorganisms (mg/L.d), d = depth of pond (m).

**Table 2:** Model calibration parameter values with summary statistics of parameters’ sensitivities.

Parameter	Description	Literature Value	Calibrated Value	References	L1 *	L2	Rank ^a^
θ	Arrhenius constant	1.01 to 1.09	1.01	[56]	1341.9	171.0	1
R_s_	ON. Sedimentation constant (d^−1^)	0.015	0.050	[46,47]	48.1	6.4	7
K_1_	Nitrosom. half sat. const. (mg/L)	0.13 to 0.15	1.26	[57]	1157.9	151.9	2
K_2_	Ammonium. half sat. const. (mg/L)	18.0	16.20	[55]	870.6	115.5	3
K_3_	Nitrate. half sat. const. (mg/L)	2.0	2.0		0	0	9
R_20_	denitrification constant (d^−1^)	0.0 to 1.0	0.26	[56]	274.6	37.3	4
Y_n_	Yield coeff. Nitrosom. (g microbes/g N)	0.13	0.163	[58]	177.4	23.1	5
µ_n_	Nitrosom. growth rate (d^−1^)	0.008	0.009		144.2	18.1	6
µ_max_	maximum growth rate at 20 °C (d^−1^)	0.1 to 0.77	0.13	[21,55]	9.5	1.3	8

* L1 = ∑S_i,j_|/*n* and L2 = $\sqrt{{S_{i,j}}^{2}/n}$ are the L1 and L2 norm, respectively, and S_i,j_ is sensitivity of parameter i for model variable j. Note: L1 is high absolute sensitivity index; L2 is used for weighted least squares estimation of parameter subset. Large differences between L1 and L2 indicate a high variability, or outliers in S_j_.

**Table 3:** Calibration and validation results for simulated concentrations with measured concentrations of ON, NH_3_, and NO_3_.

Water Quality	Nash-Sutcliffe Coefficient (NSE)		Correlation Constant (*r*)	
	Calibration	Validation	Calibration	Validation
ON	0.66	0.53	0.84	0.76
NH_3_	0.70	0.69	0.87	0.83
NO_3_	0.58	0.64	0.85	0.81

## Supplementary Materials

The following are available online at www.mdpi.com/1660-4601/14/7/765/s1, Figure S1: Collinearity index for selected 9 parameters, Table S1: List of R-FME functions used, Table S2: One-way analysis of variance (ANOVA) for ON-N. Similar results for NH_3_-N and NO_3_-N are not shown.
