# Supplementary material for: Modeling Nitrogen Dynamics in a Waste Stabilization Pond System Using Flexible Modeling Environment with MCMC
Source: Int J Environ Res Public Health. 2017 Jul 12;14(7):765. doi: 10.3390/ijerph14070765 (PMC5551203; doi:10.3390/ijerph14070765)
Supplement: Supplementary file 1 [file ijerph-14-00765-s001.pdf]

International Journal of
Environmental Research and Public Health

MDPI

Supplementary material

Modeling Nitrogen Dynamics in a Waste Stabilization Pond System Using Flexible Modeling Environment with MCMC

Hussnain Mukhtar [1], Yu-Pin Lin [1,*], Oleg V. Shipin [2] and Joy R. Petway [1]

Table S1. List of R-FME functions used [23].

Functions	Description
**sensFun**	The function is used to determine the local sensitivity of the model outputs to the parameter values, and to estimate the effect of small changes of a single parameter on model output.
**Collin**	The function estimates the approximate linear dependence of all possible parameter set (''collinearity'') by utilizing local sensitivity results (dimensionless sensitivities).
**modFit**	The function uses the minimization routines and the pseudorandom search algorithm for nonlinear model-data fitting and determine the best fit parameter set.
**modMCMC**	The function uses the Delayed Rejection (DR) and Adaptive Metropolis (AM) procedure to conduct Bayesian analysis by using a Markov chain Monte carlo (MCMC) method to obtain data-dependent probability distribution of the parameters with allowable parameter range.
**sensRange**	The function estimates the uncertainty in model output as a function of parameter probability density function, and performs global sensitivity analysis.
**modCRL**	The function determines the effect of parameter sensitivity on the mean value of a single model variable output.

Table S2. One-way analysis of variance (ANOVA) for ON-N.

**SUMMARY**						
Groups	*Count*	*Sum*	*Average*	*Variance*		
ON (influent)	49	533.09	10.87939	8.94		
ON (effluent)	49	287.75	5.872449	0.19		
ANOVA						
Source of Variation	*SS*	*df*	*MS*	*F*	*P-value*	*F crit*
Between Groups	614.2012	1	614.2012	134.4178	5.91E-20	3.940163
Within Groups	438.6572	96	4.569346			
Total	1052.858	97				

Note: Similar results for NH_3-N and NO_3-N are not shown.

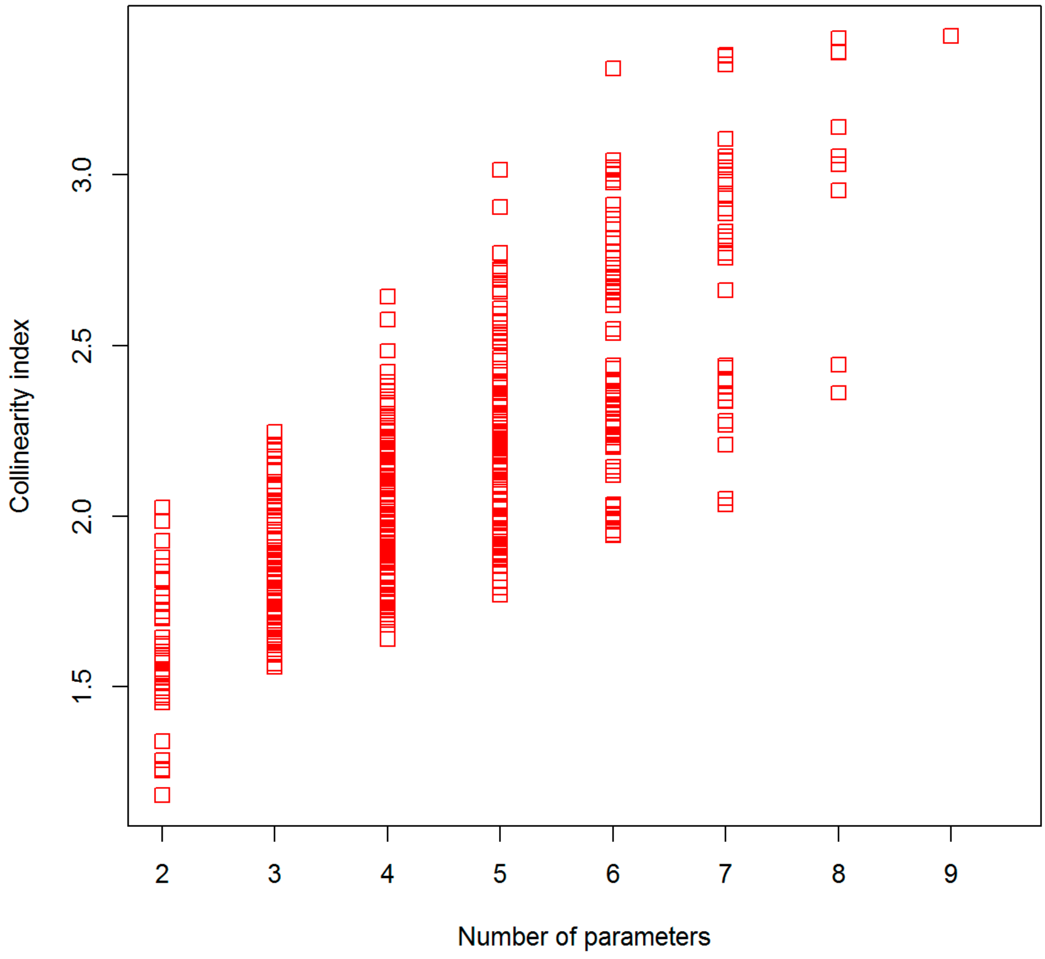

Figure S1. Collinearity index for selected 9 parameters.
